# *Brucella* NyxA and NyxB dimerization enhances effector function during infection

**DOI:** 10.1002/1873-3468.70069

**Published:** 2025-05-21

**Authors:** Lison Cancade‐Veyre, Arthur Louche, Francine C. A. Gérard, Laurent Terradot, Suzana P. Salcedo

**Affiliations:** ^1^ Laboratory of Molecular Microbiology and Structural Biochemistry, Centre National de la Recherche Scientifique UMR5086 Université de Lyon France; ^2^ Department of Pathobiological Sciences, School of Veterinary Medicine University of Wisconsin‐Madison WI USA

**Keywords:** *Brucella abortus*, dimerization, effectors, Nyx, pathogenesis

## Abstract

*Brucella abortus* is the cause of one of the most prevalent zoonoses worldwide. We have recently discovered two translocated effectors, NyxA and NyxB, that contribute to the late stages of the infectious cycle. Although their structure was solved, the importance of their interactions and dimeric states remains unknown. We found that NyxA and NyxB directly interact and that their dimerization is essential for their function during infection. We show that monomeric forms of the Nyx effectors still interact with their host cellular target, the deSUMOylase sentrin‐specific protease 3 (SENP3) but are less able than the dimers to delocalize SENP3 from the nucleoli. This study provides new insights into the intra‐ and inter‐effector molecular interactions during *Brucella* pathogenesis.

## Graphical abstract

*Brucella abortus* thrives inside cells thanks to the translocation of effector proteins that fine‐tune cellular functions. NyxA and NyxB are two effectors that destabilize the nucleolar localization of their host target, SENP3. We show that the Nyx proteins directly interact with each other and that their dimerization is essential for their function, revealing new molecular interactions contributing to *Brucella* pathogenesis.

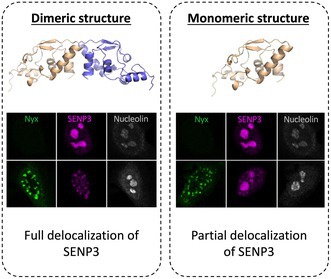

## Abbreviations

**Å**, angstrom

**BSA**, bovine serum albumin

**Da**, Dalton

**DMEM**, Dulbecco's modified Eagle medium

**DTT**, dithiothreitol

**EDTA**, éthylènediaminetétraacétique

**E‐Glu**, glutamic acid

**HBSS**, Hanks' Balanced Salt Solution

**HS**, horse serum

**IPTG**, Isopropyl ß‐d‐1‐thiogalactopyranoside

**L‐Leu**, leucine

**MOI**, multiplicity of infection

**OD**, optic density

**PBS**, phosphate‐buffered saline

**PFA**, paraformaldehyde

**SAXS**, small‐angle X‐ray scattering

**SEC‐MALS**, size exclusion chromatography coupled to multi‐angle light scattering

**SENP3**, sentrin‐specific protease 3

**TSB**, tryptic soy broth

**W‐Trp**, tryptophane

**Y‐Tyr**, tyrosine

Bacterial pathogens often rely on the translocation of effector proteins *via* dedicated secretion systems to modulate cellular functions and cause disease. The function of these effectors and their molecular interactions are essential for pathogenesis. We have recently identified two effectors, called NyxA (BAB1_0296; UniProt Q2YPD9) and NyxB (BAB1_1101; UniProt Q2YPZ9) of *Brucella abortus*, which target cytosolic and nuclear compartments during infection [1].

*Brucella* is the cause of one of the most prevalent zoonosis worldwide [2], producing a severe economic impact in livestock in endemic regions [3] and impacting wildlife [4]. *Brucella* are Gram‐negative bacteria with high pathogenic potential that rely on a type 4 secretion system to establish an intracellular multiplication niche and modulate cellular and immune functions [5, 6]. Once taken up by host cells, *Brucella* escapes destruction from the endocytic pathway and establishes a compartment suited for multiplication derived from the endoplasmic reticulum [7, 8]. During this time, *Brucella* secretes and translocates multiple effectors modulating specific host pathways to promote pathogenesis, including intracellular trafficking and the secretory pathway [9, 10, 11, 12,13], endoplasmic reticulum functions [14, 15, 16], metabolism [17, 18], and inflammatory responses [19, 20]. NyxA and NyxB have been shown to interact with the host deSUMOylase SENP3 and mediate its delocalization from the nucleoli when ectopically expressed and during infection [1]. The consequences of this delocalization are still unknown but SENP3 is necessary for full intracellular multiplication of *Brucella* [1].

The structure of NyxB was solved at 2.5 Å resolution using X‐ray crystallography, revealing it adopts a mixed α–β fold, consisting of five β‐strands and six α‐helices. A search for structural homologs using the DALI server and EMBL fold classification revealed no significant similarities, establishing NyxB as a structural prototype within its protein family [1]. The structure of NyxA was inferred by homology modeling, and both Nyx effectors were shown to form dimers in solution [1]. In this study, we aimed to investigate the molecular interactions potentially mediating the function of these effectors. We found that NyxA and NyxB can be part of the same complex in cells and that their dimer conformation is essential for SENP3 recruitment during infection.

## Materials and methods

### Cells, culture conditions, and drug treatments

HeLa‐CCL‐2 cells (RRID: CVCL_0030) and RAW 264.7 TIB‐71 macrophages (RRID: CVCL_0493) from ATCC were grown in DMEM supplemented with 10% of fetal bovine serum, at 37 °C with 5% CO_2_. Cells were routinely checked for the absence of *Mycoplasma* contamination. These cells were not authenticated as purchased directly from ATCC in 2023.

### Eukaryotic and bacterial expression vectors

The NyxA and NyxB constructs in pENTRY‐myc or HA and pET151D vectors were previously described [1]. The NyxA‐L93E and NyxB‐L97E constructs were obtained by site‐directed mutagenesis using the primers in Table 1 by site‐directed mutagenesis, following the protocol of the PrimeSTAR Max DNA Polymerase (Takara Bio).

**Table 1:** List of primers used in this study.

	Sequence 5′ → 3′	
NyxA‐L93E	Forward	GCTAAGTATGCCCGAAAGGAGGGCCTTGAGGTTAAAGAG
	Reverse	CTCTTTAACCTCAAGGCCCTCCTTTCGGGCATACTTAGC
NyxB‐L97E	Forward	TACGCCCGGACGGAGGGCCTTGAGGTT
	Reverse	AACCTCAAGGCCCTCCGTCCGGGCGTA

### *Brucella* strains, cultures, and infections

*B. abortus* 2308 was used in this study. Wild‐type and derived strains were cultured in liquid tryptic soy broth and agar. This strain is nalidixic acid resistant.

#### TEM vectors

The NyxA‐L93E and NyxB‐L97E constructs were obtained by site‐directed mutagenesis using the primers in Table 1 in pFlagTEM152‐NyxA or NyxB [1]. After verification by sequencing, plasmids were introduced into *B. abortus* 2308 by conjugation with *Escherichia coli* S17.

#### 4HA vectors

The NyxA‐L93E and NyxB‐L97E constructs were obtained by site‐directed mutagenesis of pBBR1MCS‐4‐4HA‐NyxA or NyxB [1], using the primers in Table 1. After verification by sequencing, plasmids were introduced by conjugation into *B. abortus* 2308 mutants lacking either *nyxA* or *nyxB*, respectively.

For all infections, experiments were done as described in [1]. Briefly, eukaryotic cells were seeded on glass coverslips in 24‐well plates 18 h before infection at 2 × 10^4^ cells per well. Bacterial cultures were inoculated in 2 mL of tryptic soy broth (TSB) and incubated for 18 h shaking overnight at 37 °C. Culture optical density was determined at 600 nm to dilute the cultures in order to obtain a multiplicity of infection (MOI) of 1 : 500 in the cell culture medium. Inoculated plates were centrifuged at 400 ***g*** for 10 min to initiate bacterial‐cell contact and incubated for 1 h at 37 °C and 5% CO_2_ for the infection to start. Cells were then washed three times with DMEM and incubated with complete media supplemented with gentamycin (50 μg·mL^−1^) to kill extracellular bacteria. After 1 h, the gentamycin concentration was reduced to 10 μg·mL^−1^. At the different time points, coverslips were fixed for immunostaining as described below.

### TEM1 translocation assay

RAW 264.7 cells were seeded in 96‐well plates at 0.5 × 10^4^ cells per well overnight. Cells were inoculated at an MOI of 500 and centrifuged at 400 ***g*** for 10 min at 4 °C to prevent phagocytosis and synchronize the infection. Infected cells were then incubated for 45 min at 37 °C with 5% CO_2_. IPTG at 1 mm was maintained throughout the infection to induce the expression of the TEM fusions. After the 45‐min incubation, cells were washed with DMEM and incubated with media containing gentamycin (50 μg·mL^−1^) for 1 h, when the antibiotic concentration was reduced to 10 μg·mL^−1^. At 24 post‐infection, CCF2 mix was prepared as described in the Life Technologies (Carlsbad, CA, USA) protocol and added, along with probenecid 2.5 mm (to reduce cellular export of CCF2). Cells were incubated for 2 h at room temperature in the dark. Cells were washed with PBS, fixed using 3.2% PFA, and analyzed immediately by confocal microscopy (Nikon AR1‐Si^+^, Tokyo, Japan).

### Protein expression and purification

The Nyx mutants were purified using the same protocol as previously described for the wild‐type NyxA and NyxB [1]. *E. coli* BL21‐DE3‐pLysS thermocompetent bacteria were transformed with the different expression vectors and grown in lysogenic broth (LB) media to OD_600_ = 0.6. Their expression was induced with 1 mm IPTG at 37 °C for 3 h with shaking. Cells were then collected by centrifugation at 5000 ***g*** for 15 min and resuspended in lysis buffer 20 mm Tris pH 8, 150 mm NaCl, 5% glycerol, 1% Triton X‐100, 5 mm β‐mercaptoethanol. Antiprotease EDTA‐Free cocktail and 30 U·mL^−1^ benzonase were added. Sonication was used for cell disruption and debris was removed by centrifugation at 20 000 ***g*** for 30 min at 4 °C. All the recombinant proteins were purified by chromatography using a Nickel‐loaded Hitrap Chelating HP column (GE Healthcare, Chicago, IL, USA). Unbound material was washed using a solution composed of Tris 20 mm pH 8, NaCl 300 mm, 25 mm Imidazole, 5 mm β‐mercaptoethanol, and 10% glycerol. This was followed by an additional wash with 2 column volumes of 1 m NaCl. Elution of each Nyx protein was achieved using a 25–500 mm imidazole gradient over 8 column volumes, and by pooling all the peak fractions. Finally, the His tag was cleaved with the TEV protease in 1 mm DTT and 0.5 mm EDTA in overnight dialysis buffer composed of 20 mm Tris pH 8 and 150 mm NaCl.

An additional purification step was performed by size exclusion chromatography (Superdex 200 HiLoad 16/600, GE Healthcare) equilibrated in 20 mm Tris pH 8, 150 mm NaCl, and 5% glycerol. Samples were concentrated on a 5 kDa Vivaspin concentrator, and their purity was verified by SDS/PAGE.

### Size exclusion multi‐angle light scattering

These methods were previously described in [21]. The size exclusion chromatography (SEC) was performed with a Superdex S200 Increase 10/300 GL or Superdex 75 Increase 10/300 GL (Cytiva, Marlborough, MA, USA) equilibrated with a 20 mm Tris pH 8 buffer, containing 150 mm NaCl, and 5% glycerol. A flow rate of 0.5 mL·min^−1^ was used at 20 °C. Proteins were injected at a concentration of 0.4 to 11 mg·mL^−1^ (50 μL). Online multi‐angle laser light scattering (MALS) detection was performed with a DAWN‐HELEOS II detector (Wyatt Technology Corp., Santa Barbara, CA, USA) using a laser emitting at 690 nm. The protein concentration was measured online using differential refractive index (RI) measurements with an Optilab T‐rEX detector (Wyatt Technology) and an RI increment dn/dc of 0.185 mL·g^−1^. The weight‐averaged molecular mass (Mw) was calculated using the astra software (Wyatt Technology Corp.).

### Transfection

All cells were transiently transfected using JetOPTIMUS (Polyplus, Illkirch, France) according to the manufacturer's instructions for 18 h.

### Immunofluorescence microscopy

Coverslips were washed twice with PBS, fixed at the desired time points with either AntigenFix (MicromMicrotech France, Brignais, France) or PFA 3.2% (Electron Microscopy Sciences, Hatfield, PA, USA) for 20 min and then washed again four times with PBS. Coverslips were then incubated with PBS containing 0.5% Triton X‐100 for 10 min to permeabilize cells, and blocking was done with 0.3% triton, 2% bovine serum albumin (BSA), and 10% horse serum (HS) in PBS for 20 min.

Of note, detection of translocated HA‐tagged effectors was achieved by permeabilizing cells with 0.3% triton in PBS for 10 min and minimize labeling of intravacuolar proteins. Blocking was done for 15 min with 2% BSA and 10% HS in PBS but in the absence of triton.

All primary antibodies (Table 2) diluted in 2% BSA and 10% HS were incubated at 4 °C overnight in a humid chamber. The coverslips were washed twice in PBS and incubated for an additional 1 h at room temperature in the dark and in a humid chamber with the mix of secondary antibodies. Finally, the coverslips were washed twice in PBS and once in ultrapure water prior to mounting on a slide with ProLongGold (Life Technologies). Immunolabeled cells were imaged with a Confocal Zeiss inverted laser‐scanning microscope LSM980 NLO and Leica SP5 and analyzed using imagej.

**Table 2:** List of antibodies used in this study.

Antibody	Species	Dilution WB	Dilution IF	References
Anti‐GFP	Rabbit	1/5000		Amsbio (Abingdon, England) #TP401
Anti‐HA	Rat		1/100	Roche (Basel, Switzerland) #11867423001
Anti‐HA	Rabbit	1/2000		Cell Signaling (Danvers, MA, USA) #C29F4
Anti‐Myc	Mouse	1/1000	1/100	Developmental Studies Hybridoma Bank (Iowa, IA, USA) – DSHB #9E10
Anti‐Nyx	Rabbit	1/2000		Eurogentec [1]
Anti‐Nucleolin	Mouse		1/100	Invitrogen (Waltham, MA, USA) #39‐6400
Anti‐SENP3	Rabbit	1/1000	1/400	Cell Signaling #5591

### Co‐immunoprecipitation

HeLa cells were cultured in 100 × 20 mm cell culture‐treated dishes at 1.5 × 10^6^ cells per dish overnight and transiently transfected with the JetOPTIMUS (Polypus). A total of 10 μg of DNA was added per plate and incubated at 37 °C, 5% CO_2_ for 18 h. To minimize toxicity, the media was replaced 4 h after transfection. After 18 h, plates were placed on ice and washed twice with ice‐cold PBS. Cells were collected with a cell scraper and centrifuged at 80 ***g*** at 4 °C for 5 min. Cell lysis and processing for co‐immunoprecipitation were done as described with the GFP‐Trap kit (Chromotek, Rosemont, IL, USA) or as described for Pierce™ Anti‐HA Magnetic Beads (Thermo Fisher, Waltham, MA, USA).

### Pull‐down assays

Pull‐down assays were done as described previously [1]. Briefly, with two purified proteins, 50 μg of the His‐tagged recombinant protein was incubated with the untagged one during 2 h at 4 °C. Samples were then incubated within a gravity flow column (Agilent, Santa Clara, CA, USA) containing 80 μL Ni‐NTA agarose beads (Macherey‐Nagel, Düren, Deutschland) during 1 h. The column was prewashed in water and pre‐equilibrated in equilibrium buffer 20 mm Tris/HCl pH 7.5, 250 mm NaCl. After incubating with the protein mix, the column was washed three times in equilibrium buffer supplemented with 25 mm imidazole and three times in equilibrium buffer alone. Proteins were eluted in equilibrium buffer supplemented with 500 mm imidazole. Results were visualized after separation by SDS/PAGE by Coomassie staining.

### Quantification of SENP3 localization

Colocalization analysis for the transfected HeLa cells was performed with a custom imagej developed in [1]. Briefly, the plugin segments the nuclei and the nucleoli of the cells in each image, classifying the cells into two classes according to the intensity of HA‐NyxA/NyxB. A measure of the Pearson correlation coefficients between the signal of SENP3 and the nucleolin as well as between the signals of SENP3 and HA‐NyxA/NyxB or mutants was obtained. For each nucleus, the ratio between the mean intensity of the SENP3 signal in the nucleoli and the mean intensity of the SENP3 signal outside the nucleoli was also calculated. A single experiment was done blinded to confirm unbiased image acquisition. A similar plugin was used for infected cells, which is also described in [1] and calculates the Pearson correlation coefficients between the signal of SENP3 and the nucleolin found in each nucleus of an infected cell, detected with the DSRed fluorescence of the bacteria. Once a normal distribution was confirmed using the Shapiro–Wilk normality test, a one‐way ANOVA test with Tukey's correction for multiple comparisons was performed. For analyses involving two independent variables, a two‐way ANOVA test was performed.

## Results

### NyxA and NyxB interact *in vitro* and *in cellulo*

**Fig. 1:**
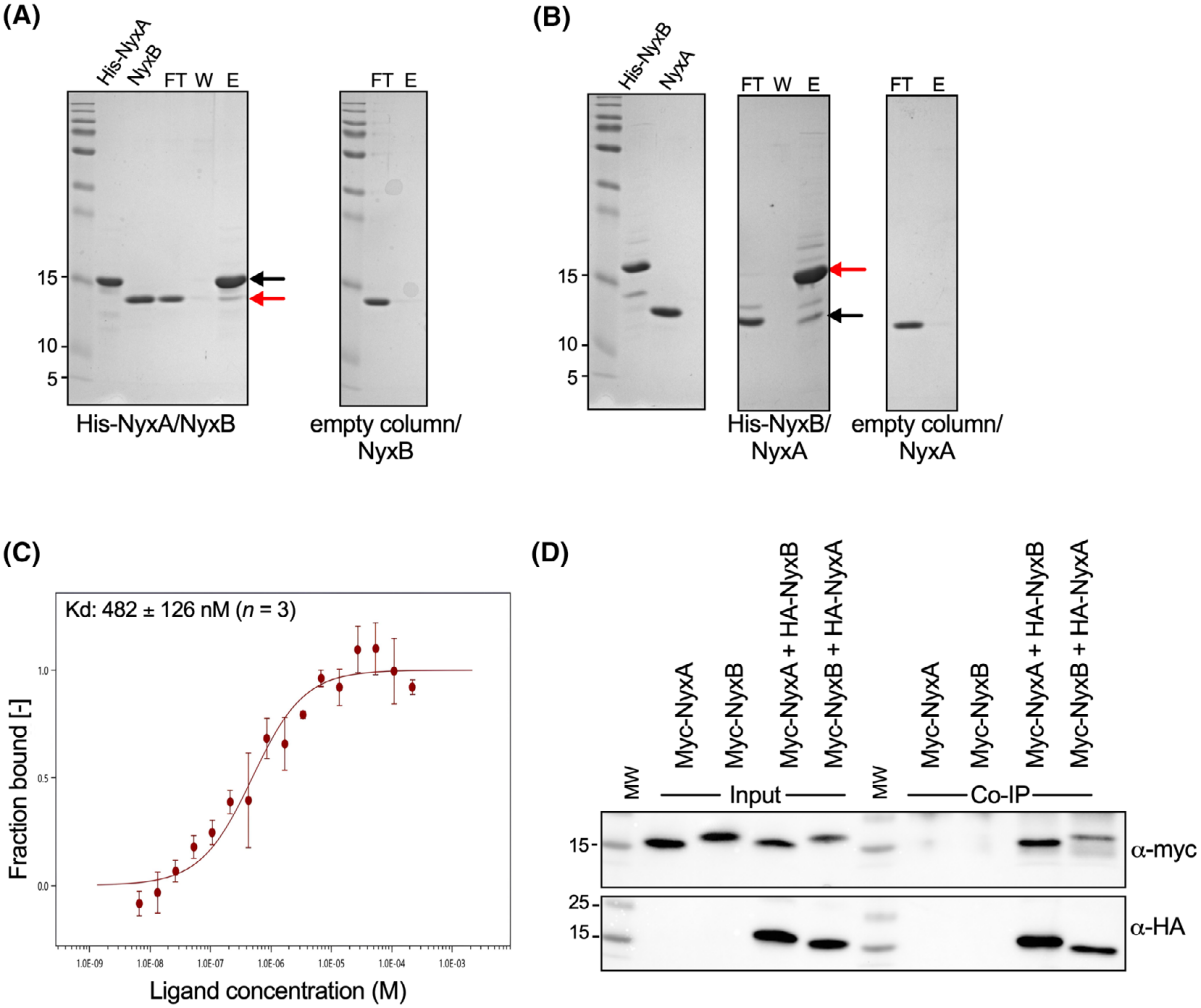
*In vitro* and *in cellulo* interaction between NyxA and NyxB. (A) Pull‐down using purified NyxB against His‐NyxA immobilized on a Ni NTA resin or the inverse (B) His‐NyxB immobilized. For both, an empty column was used as a control for non‐specific binding. Interactions were visualized with coomassie blue stained gels (*N* = 3, with one representative each shown). The flowthrough (FT), wash (W), and elution (E) fractions are shown for each sample, and the molecular weights are indicated (kDa). Eluted NyxA and NyxB are indicated with black and red arrows, respectively. In both (A) and (B), black arrows indicate NyxA, and red arrows indicate NyxB. (C) Microscale thermophoresis measuring the fraction of 20 nm of purified NyxA labeled with kit protein labeling RED‐NHS binding to increasing concentrations of NyxB (6.67 nm to 219 μm). Data correspond to means ± standard deviations of three independent experiments. The obtained Kd is indicated. (D) Co‐immunoprecipitation (co‐IP) assay from cells expressing NyxA or NyxB tagged with HA or Myc using anti‐HA‐magnetic beads. This experiment was repeated three times, and a representative is shown. The input and co‐IP fractions were revealed using anti‐Myc (top) and anti‐HA antibodies (bottom). Molecular weights are indicated (kDa).

We had previously shown that NyxA and NyxB can efficiently delocalize SENP3 from the nucleoli when individually expressed ectopically in host cells [1]. However, when co‐expressed, they fully colocalize [1] suggesting they could be potentially part of the same complex. To determine if these two effectors can directly interact, we purified them and carried out pull‐down experiments *in vitro*. We found that His‐NyxA could pull down purified untagged NyxB (Fig. 1A) and vice versa (Fig. 1B). The identity of the visualized bands was verified by mass spectrometry. Microscale thermophoresis also confirmed this interaction and determined a *K*_D_ of 482 ± 126 nm (Fig. 1C), indicative of a rather low affinity complex.

To determine if NyxA and NyxB could also be part of the same complex *in cellulo*, we ectopically expressed them taking advantage of different tagged versions of these proteins and carried out a co‐immunoprecipitation experiment. Using HA magnetic beads, we found that myc‐NyxA could co‐immunoprecipitate with HA‐NyxB and, reciprocally, myc‐NyxB could immunoprecipitate with HA‐NyxA (Fig. 1D). Together, these results suggest that NyxA and NyxB interact together and are part of the same complex in cells.

Due to the low amounts of translocated Nyx during infection, we were not able to test this interaction directly in infected cells using biochemical assays. Nonetheless, this hypothesis is consistent with the fact that deletion of both *nyxA* and *nyxB* results in the same phenotype as deletion of *nyxA* and *nyxB* individually, in terms of SENP3 delocalization from the nucleoli, showing that there is no additive effect of the two gene deletions [1]. Furthermore, translocated NyxA and NyxB have equivalent cellular localizations in infected cells (co‐localizing with the same cellular markers) as observed using proximity ligation assay and HA/Flag tags [1]. Therefore, we conclude that NyxA and NyxB are likely part of the same complex in infected cells, responsible for the SENP3 delocalization observed at late stages of the infection.

### Identification of key amino acids on the Nyx dimerization surface

**Fig. 2:**
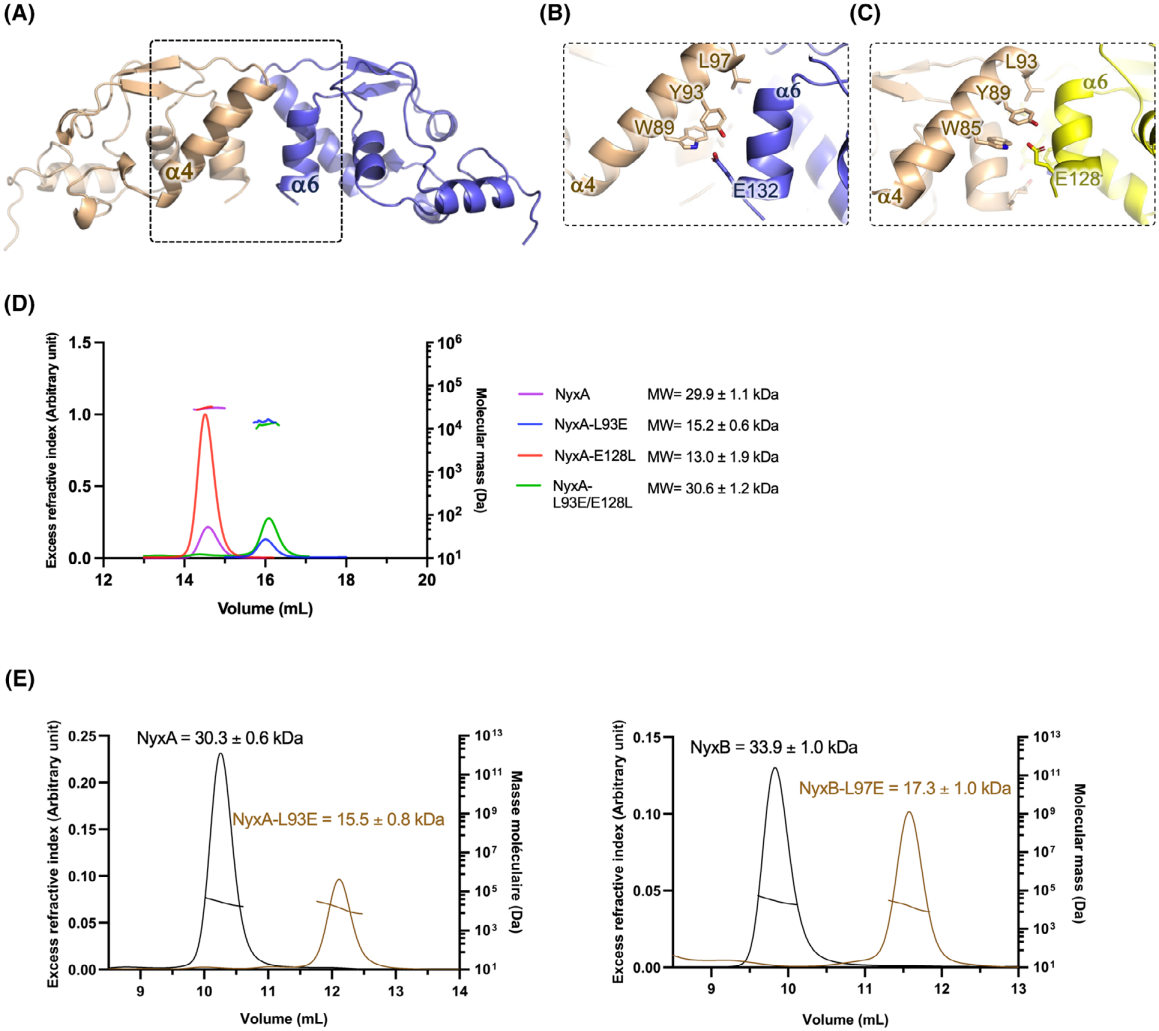
Analysis of the dimer interface and identification of key amino acids involved. (A) Structure of the NyxB dimer. (B) Close‐up view of the residues involved in the homodimer interface of NyxB. (C) Close‐up view of the residues involved in the homodimer interface of the NyxA model. pymol was used to make the figures for (A), (B), and (C). (D) Size Exclusion Chromatography and Multi‐Angle Light Scattering (SEC‐MALS) of NyxA and mutants. The elution was monitored online using multi‐angle laser light scattering and refractometry. The curves show the chromatograms monitored by differential refractive index measurement. The horizontal lines above the peaks indicate the molecular mass across the elution peak calculated from static light scattering, and the numbers indicate the weight‐averaged molecular mass (kDa) with standard deviations. Data are representative of two independent experiments. (E) SEC‐MALS of NyxA and NyxB (black lines) and the monomeric mutants of each (cream lines). The average molecular weights and standard deviations determined are indicated. Data are representative of two independent experiments.

The structure of the NyxB revealed a dimer organization, which was also visualized by small‐angle X‐ray scattering (SAXS) and size exclusion chromatography coupled to multi‐angle light scattering (SEC‐MALS) for both NyxA and NyxB [1]. We therefore wanted to investigate whether dimer formation contributed to the function of these effectors. To begin addressing this, we first identified key amino acids involved in dimer formation. NyxB dimer relies on reciprocal interactions between α4 of one subunit and α6 of the other subunit and between the two α4–β4 loops (Fig. 2A) NyxB residues W89, Y93, L97 from α4 and E132 from α6 are interacting together predominantly *via* hydrophobic interactions (Fig. 2B). By analyzing the dimer of NyxA homology model previously generated [1], we found that these residues and interactions were conserved in NyxA, with the corresponding residues being W85, Y89, L93, and E128 (Fig. 2C). To facilitate mutagenesis, we selected to mutate L93 and E128 that are located at the C‐termini of helix α4 and α6, respectively, instead of W85 and Y89 which are positioned in the middle of α4 (Fig. 2B). To disrupt the dimer interface, we generated the following NyxA mutants by site‐directed mutagenesis: L93E, E128L, and the double mutant. We were able to purify the three mutants and analyzed their oligomeric states by size exclusion chromatography coupled to SEC‐MALS using a Superdex 200 Increase 10/300 GL. These experiments revealed that the double mutant and NyxA‐L93E eluted as monomers, whereas mutation of E128L had no impact on the oligomeric state of the eluted protein (Fig. 2D). To confirm that this leucine was essential for dimer formation, we also generated the equivalent mutant in NyxB (NyxB‐L97E) and analyzed both NyxA and NyxB wild‐type and mutant proteins by SEC‐MALS using this time a Superdex 75 Increase 10/300 GL giving improved separation in the lower molecular weight range. Introduction of the L93E mutation into NyxA and L97E into NyxB resulted in a change of the elution volume and the molecular mass decreased to 15 and 17 kDa, respectively, in contrast to the respective wild‐type proteins (Fig. 2E). These results confirm that mutation of this leucine dissociates the dimer, and both Nyx mutants are monomeric in solution. Altogether, our results clearly establish that the dimer observed in the NyxB structure is the correct one and is conserved in NyxA but also that these protein mutants are stable as monomers.

### Monomeric Nyx still targets the nucleus and is part of the same complex as SENP3 in host cells

**Fig. 3:**
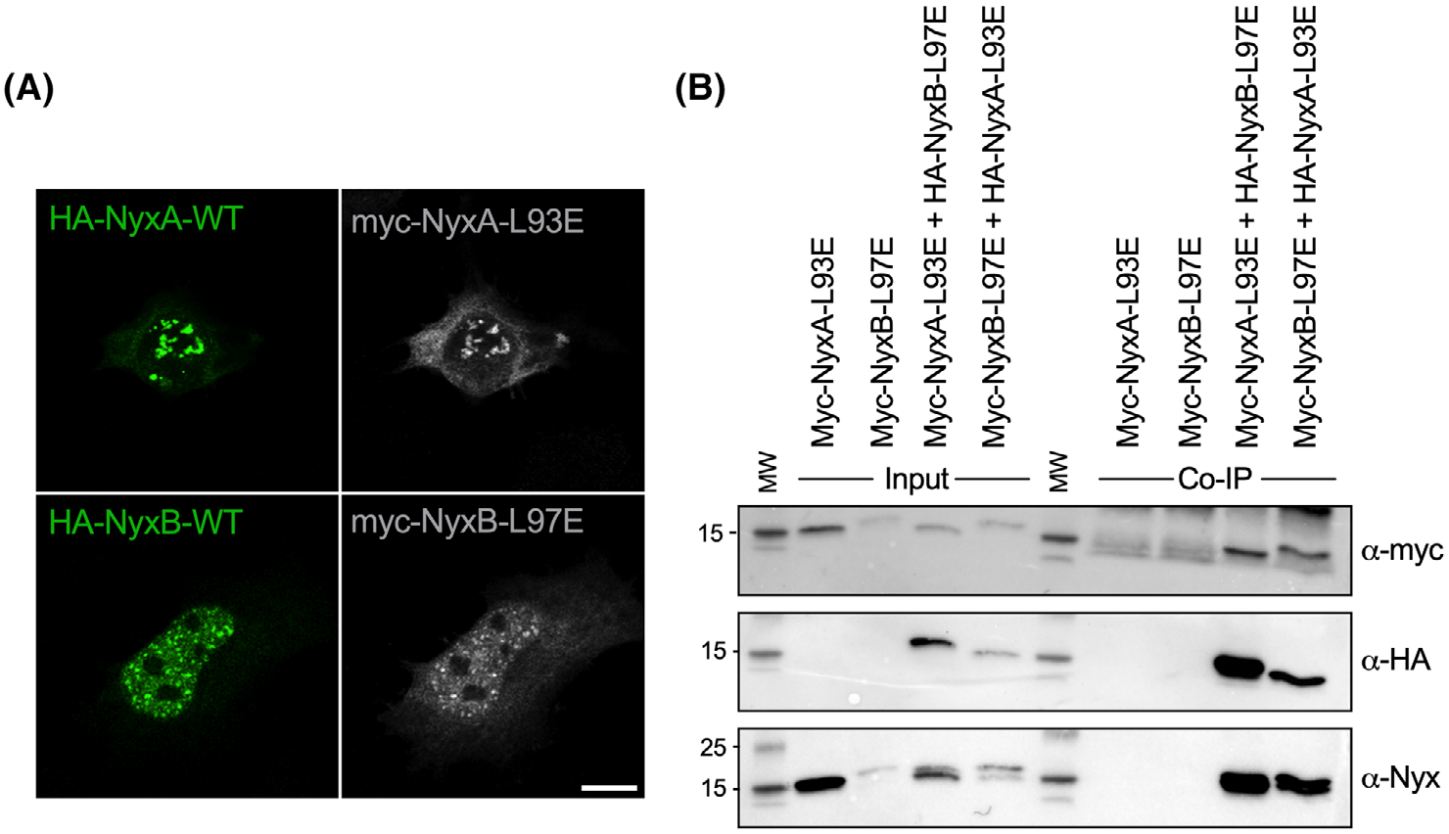
Monomeric NyxA and NyxB still target the nucleus and are part of the same complex. (A) Representative confocal images of co‐transfected epithelial cells showing in the top panel the localization of HA‐NyxA (green) in relation to myc‐NyxA‐L93E (gray) and, in the bottom panel, HA‐NyxB (green) in relation to myc‐NyxB‐L97E (gray) (*N* = 1). Scale bar corresponds to 5 μm. (B) Co‐immunoprecipitation (co‐IP) assay from cells expressing NyxA‐L93E or NyxB‐L97E tagged with HA or Myc. The input and co‐IP fractions were revealed using an anti‐Myc (top), an anti‐HA (middle), and an anti‐Nyx antibody (bottom). Molecular weights are indicated (kDa). Figure corresponds to a representative of three independent experiments.

To determine the impact of preventing Nyx dimerization, we ectopically expressed myc‐tagged monomeric NyxA‐L93E and NyxB‐L97E and visualized their localization in relation to the corresponding wild‐type Nyx. As previously reported for the wild‐type NyxA/B, the monomeric mutants still accumulated in the nucleus, in punctate structures (Fig. 3A). Consistently, monomeric NyxA‐L93E and NyxB‐L97E still co‐immunoprecipitated when ectopically expressed, suggesting they are part of the same complex in host cells (Fig. 3B). As non‐specific bands were detected with the anti‐myc antibody, we confirmed the absence of monomeric myc‐Nyx binding to the HA‐beads in the control samples using an anti‐Nyx antibody, which recognizes both NyxA and NyxB (Fig. 3B, bottom image).

**Fig. 4:**
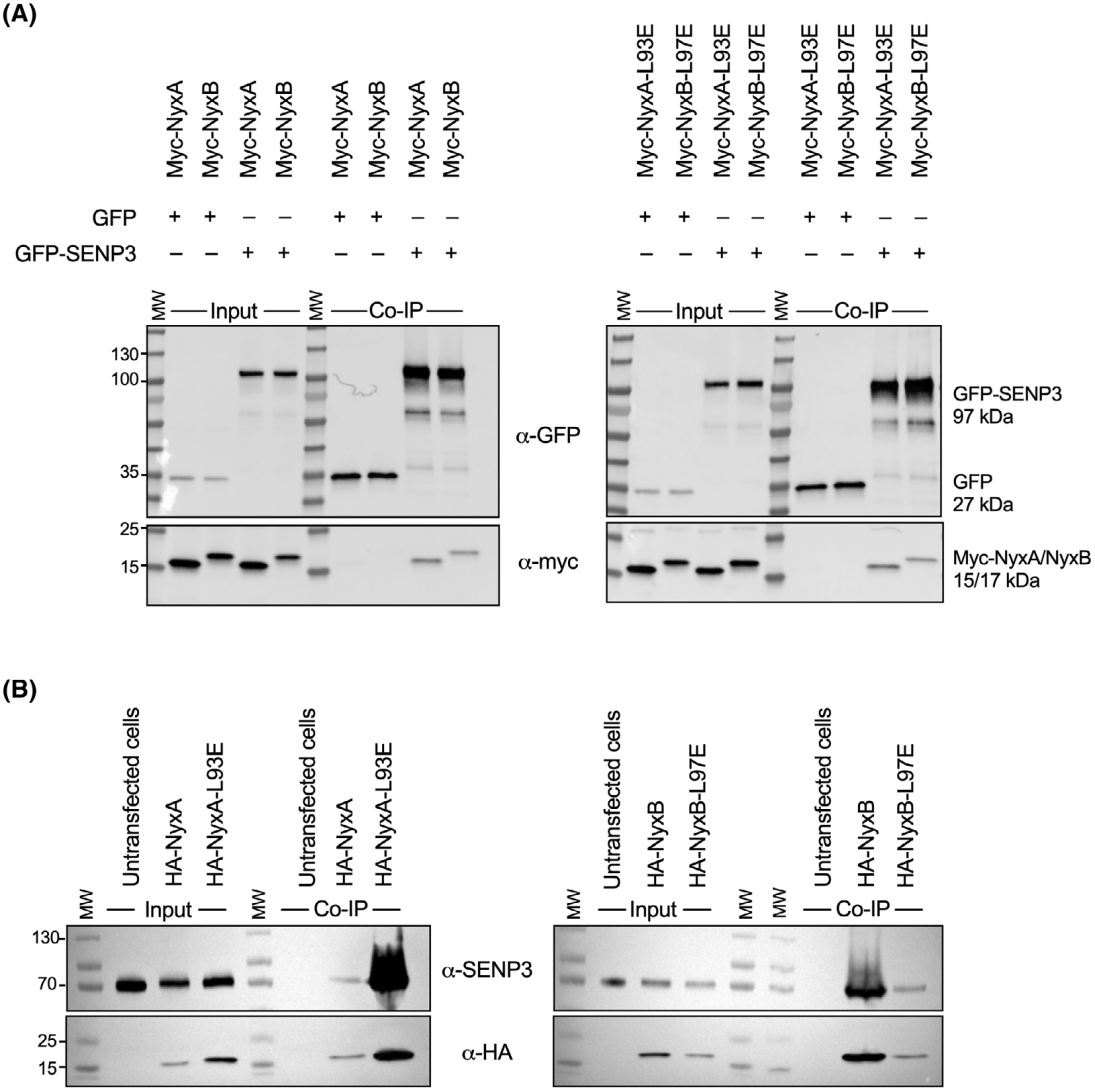
Monomeric NyxA and NyxB still interact with SENP3. (A) Co‐immunoprecipitation (co‐IP) assay using GFP‐Trap, incubated with lysates from cells expressing GFP‐SENP3 and Myc‐NyxA, NyxB, NyxA‐L93E, or NyxB‐L97E. GFP was used as a control for non‐specific binding. The inputs and co‐IP elutions were revealed using an anti‐Myc antibody (bottom) and an anti‐GFP antibody (top). Molecular weights are indicated (kDa). *N* = 3 (representative shown). (B) Co‐immunoprecipitation (co‐IP) assay using anti‐HA magnetic beads, incubated with lysates from cells expressing HA‐NyxA, HA‐NyxA‐L93E, HA‐NyxB, or HA‐NyxB‐L97E. Cells transfected with the HA empty vector were used as a control for nonspecific binding. The inputs and co‐IP elutions were revealed using an anti‐HA antibody (bottom) and an anti‐SENP3 antibody (top). Molecular weights are indicated (kDa). *N* = 3 (representative shown).

To assess if the monomeric Nyx mutants were still able to interact with their previously identified cellular target SENP3, we first ectopically expressed myc‐tagged versions of each Nyx and co‐expressed them with GFP‐SENP3 or GFP alone as a control for nonspecific binding. Our results showed efficient co‐immunoprecipitation of NyxA and NyxB as well as the monomeric mutants with GFP‐SENP3, but not with GFP alone (Fig. 4A). To confirm these results, we also carried out endogenous co‐immunoprecipitation. Both monomeric mutants of NyxA and NyxB were able to co‐immunoprecipitate endogenous SENP3, as previously observed for the wild‐type (Fig. 4B). Together, our results confirm that monomeric NyxA and NyxB are still in the same complex as SENP3 in host cells.

### Dimerization of NyxA/NyxB enhances the recruitment of SENP3

**Fig. 5:**
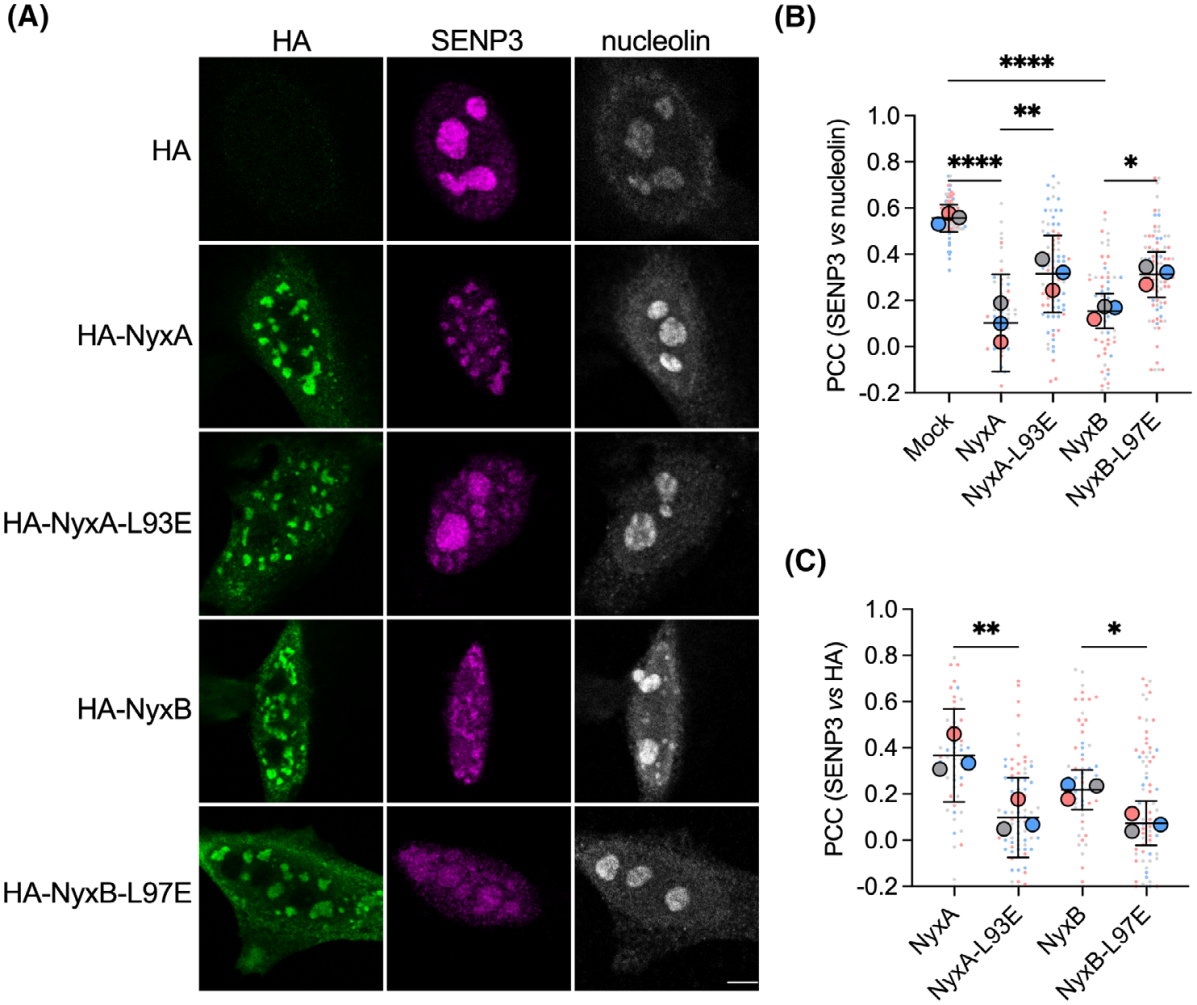
NyxA and NyxB dimerization enhances SENP3 recruitment when ectopically expressed and during infection. (A) Representative confocal microscopy images of HeLa cells expressing the HA empty vector, HA‐NyxA, HA‐NyxA‐L93E, HA‐NyxB, and HA‐NyxB‐L97E. Nucleolin (gray), SENP3 (magenta), and HA (green) were revealed with specific antibodies. The experiment was repeated three times, and images were quantified in (B). Scale bar corresponds to 5 μm. (B) Quantification of the Pearson correlation coefficient of SENP3 versus nucleolin or (C) SENP3 versus HA (see Materials and methods for plugin description). Data are represented as means ± 95% confidence intervals from three independent experiments. Each experiment is color coded, and all events counted are shown. Data were analyzed using one‐way ANOVA. Not all comparisons are shown. All the cells quantified are shown in the format of Super Plots, with each color representing an independent experiment and its corresponding mean (*N* = 3). *Indicates *P* < 0.05; **P < 0.01, and ****P < 0.0001.

**Fig. 6:**
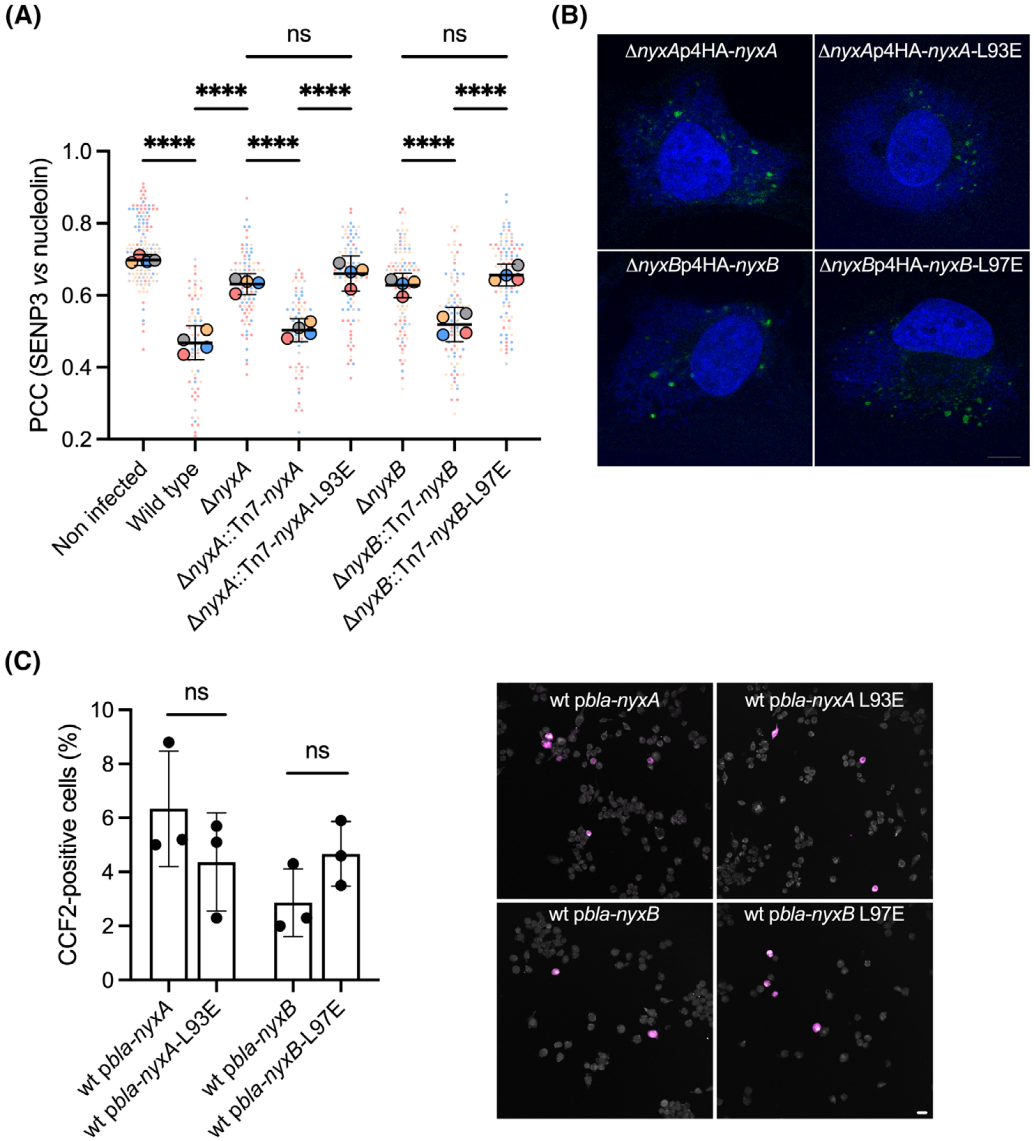
NyxA and NyxB dimerization is essential for SENP3 delocalization in infected cells. (A) Quantification of the Pearson correlation coefficient of SENP3 versus nucleolin in cells infected with the different *B. abortus* strains for 48 h. Strains used include wild‐type, mutants lacking *nyxA* or *nyxB*, or respective complemented strains with either NyxA and NyxB or monomeric NyxA‐L93E or monomeric NyxB‐L97E. Data are represented as means ± 95% confidence intervals from four independent experiments. Each experiment is color coded, and all events counted are shown. Data were analyzed using one‐way ANOVA. ****Indicates *P* < 0.0001 and ns, nonsignificant. Not all comparisons are shown. All the cells quantified are shown in the format of Super Plots, with each color representing an independent experiment and its corresponding mean. (B) Confocal images of HeLa cells infected with *B. abortus* Δ*nyxA* or Δ*nyxB* expressing either 4HA‐NyxA or 4HA‐NyxB, respectively, in comparison with the respective monomeric mutants, 4HA‐NyxA‐L93E and 4HA‐NyxB‐L97E. Bacteria are visualized with DAPI and the translocated 4HA fusions with anti‐HA antibody (green). This imaging was done once. Scale bar corresponds to 10 μm. (C) Analysis of translocation of NyxA, NyxB, and monomeric mutants fused with the TEM‐1 lactamase after 24 h of infection of RAW 264.7 macrophages. Data correspond to means ± SD (*N* = 3). One‐way ANOVA was used for comparisons. Representative images are shown, with CCF2‐positive cells in magenta, and scale bar corresponds to 20 μm.

We next investigated if the monomeric mutants of NyxA/B still recruited SENP3, resulting in its accumulation outside the nucleoli. Quantification by microscopy suggests that the monomeric mutants are less efficient in delocalizing SENP3 from the nucleoli (Fig. 5A,B) and recruiting it to the Nyx subnuclear aggregates (Fig. 5C) when ectopically expressed in host cells. In view of these results, we tested if SENP3 recruitment was impacted in infected cells, taking advantage of complementing vectors either expressing wild‐type or monomeric versions of NyxA and NyxB. After 48‐h postinfection, expression of the monomeric versions of NyxA and NyxB did not complement the respective deletion phenotypes (Fig. 6A), confirming Nyx dimerization is essential for SENP3 delocalization during infection. Importantly, the translocation of NyxA/B in infected cells was not affected by mutation of the residue involved in dimer formation as visualized using the 4HA‐tagged versions of these monomeric mutants (Fig. 6B) or quantified by the TEM β‐lactamase translocation assay (Fig. 6C).

## Discussion

We have used the crystal structure of the NyxB dimer as a template to study the role of Nyx protein dimerization. Our mutagenesis and biochemical study demonstrate that the dimer observed in the NyxB structure is the correct one, as mutation of the interface impacts dimer stability. Moreover, we show that the dimer assembly mode is conserved in the two Nyx proteins. Using ectopic expression and infection assays, we demonstrate that Nyx dimerization plays an important role in their function. We cannot at this stage conclude if heterodimers or heterotetramers are forming during infection. Additional *in situ* biochemical characterization needs to be undertaken during infection but we require improved approaches to monitor translocated effectors, under native conditions. Our data suggest that the dimer interface must be distinct from the NyxA‐NyxB interaction interface. Indeed, in the crystal structure of NyxB, two different dimers could be observed that we named dimer 1 and dimer 2 in the original publication [1]. While our previous study and the data presented here clearly demonstrate that dimer 1 is the homodimeric form of NyxB, it is possible that a dimer 1 of NyxA assembles with a dimer 1 of NyxB *via* the second interface we previously identified [1]. In host cells during infection, these two conformations could be relevant, one of them enabling dimerization and the other enabling NyxA‐NyxB heterotetramerization. Nyx heterotetramer formation could potentially increase the efficiency of SENP3 recruitment or the formation of larger complexes with other cellular targets yet to be identified.

SENP3 delocalization was used as a measure of Nyx function during infection. Although the consequences of this interaction remain unclear, SENP3 is important for intracellular multiplication at the late stage of the intracellular *Brucella* cycle. Despite observing that the monomeric forms of NyxA and NyxB were still capable of interacting with SENP3 *in vitro* and when ectopically expressed in cells, the efficiency of SENP3 recruitment was significantly reduced. This likely explains the result in infected cells where almost no delocalization of SENP3 was observed. It is possible that the monomers have a reduced interaction with SENP3 not detectable by co‐immunoprecipitation. It is also possible that additional interactions are occurring during infection, for example, with other effectors, that are perturbed by the loss of the Nyx dimeric state. Consistently, deletion of both NyxA and NyxB is not sufficient to return to a noninfected state in terms of SENP3 localization and does not impact intracellular multiplication as observed in SENP3‐depleted cells, suggesting additional effectors may be involved. We cannot exclude that the monomeric NyxA‐L93E and NyxB‐L97E mutations have additional effects besides impacting dimer formation, which would perturb their functions in host cells.

These results highlight the importance of the molecular interactions taking place between the Nyx effectors and how their dimerized state contributes to their function during infection.

## Author contributions

LC‐V conceived all experiments and generated all the data except for Fig. 1A–C, interpreted results, and wrote the manuscript. AL performed the experiments in Fig. 1A–C. FCAG contributed to the identification of the amino acids likely needed for dimer formation and the MALS. LT identified the dimer interface and conceived some of the experiments, contributed to data analysis, and manuscript writing. SPS supervised the project, contributed to data analysis, and wrote the manuscript. All authors read and corrected the manuscript and agree with the contents.

## Peer review

The peer review history for this article is available at https://www.webofscience.com/api/gateway/wos/peer‐review/10.1002/1873‐3468.70069.

## Data Availability

The raw data (uncropped blots and gels) that support the findings of this study are openly available in DRYAD at https://doi.org/10.5061/dryad.fxpnvx14s.
